# MYB transcription factor PdMYB118 directly interacts with bHLH transcription factor PdTT8 to regulate wound-induced anthocyanin biosynthesis in poplar

**DOI:** 10.1186/s12870-020-02389-1

**Published:** 2020-04-20

**Authors:** Haihai Wang, Xiaoqing Wang, Chunyan Yu, Cuiting Wang, Yanli Jin, Hongxia Zhang

**Affiliations:** 1 National Key Laboratory of Plant Molecular Genetics, Shanghai Institute of Plant Physiology and Ecology, Chinese Academy of Sciences, 300 Fenglin Road, Shanghai, 200032 China; 2 Forestry and Pomology Research Institute, Shanghai Academy of Agriculture Sciences, 1000 Jinqi Road, Shanghai, China; 3 College of Agriculture, Ludong University, 186 Hongqizhong Road, Yantai, 264025 China; 4 The Key Laboratory of Molecular Module-Based Breeding of High Yield and abiotic Resistant Plants in the Universities of Shandong, Ludong University, 186 Hongqizhong Road, Yantai, 264025 China

**Keywords:** Anthocyanin, JAZ1, PdMYB118, PdTT8, Poplar

## Abstract

**Background:**

R2R3-MYB transcription factors (TFs) play important roles in plant growth and development, and response to biotic and abiotic stresses. However, their regulatory mechanisms in wound-induced anthocyanin biosynthesis in woody plants are largely unknown.

**Results:**

In this work, we report that expression of anthocyanin biosynthesis genes (*ABGs*) were activated by PdMYB118, a MYB TF encoding gene from *Populus deltoids*, and the activation of PdMYB118 was significantly enhanced by PdTT8, a bHLH protein, through its direct interaction with PdMYB118. *PdMYB118* and some *ABGs* were evidently induced by wound induction and methyl jasmonate (MeJA) treatment. Overexpression of *PdMYB118* promoted anthocyanin accumulation in transgenic poplar upon wound induction. Furthermore, a poplar JASMONATE ZIM-domain (JAZ) protein, PtrJAZ1, repressed the transcriptional function of PdMYB118/PdTT8 complex by binding to PdTT8, and wound stimulated the biosynthesis of jasmonic acid (JA) and the degradation of PtrJAZ1.

**Conclusions:**

Based on these observations, we proposed that PtrJAZ1 degradation triggered the expression of *ABGs*, leading to increased biosynthesis of anthocyanins in the wounded leaves of transgenic poplar. Therefore, our findings not only illustrate the crucial role of PdMYB118 in wound-induced anthocyanin biosynthesis in poplar, but also provide a molecular basis for the genetic engineering of colorful tree species.

## Background

As the major pigments, anthocyanins not only provide colors to plant flowers and fruits [1], but also function in plant resistance to different biotic and abiotic stresses [2–12]. In plants, anthocyanins are biosynthesized via the specific branch of flavonoid pathway [1, 13, 14]. In poplar, the biosynthesis of anthocyanins is catalized by a series of enzymes, including chalcone synthase (CHS), chalcone isomerase (CHI), flavanone 3-hydroxylase (F3H), flavanone 3′-hydroxylase (F3’H), flavonoid 3′5’-hydroxylase (F3’5’H), dihydroflavonol 4-reductase (DFR) and anthocyanidin synthase (ANS) [7, 8]. The expression of anthocyanin biosynthetic genes (*ABGs*) are regulated by the MBW complexes, which is composed of two kinds of transcriptional factors (TFs), R2R3-MYB and basic helix-loop-helix (bHLH), and WD40-repeat proteins [15–17]. In *Arabidopsis*, the MBW complexes are composed of the R2R3-MYB factors PAP1, PAP2, MYB113 and MYB114, the bHLH factors TT8, GL3 and EGL3, and a WD40 protein TTG1 [16]. So far, similar MBW complexes have been identified in different plant species [13, 18–22].

JA is one of the essential phytohormones which plays important functions in the protection of plants from various biotic and abiotic stresses [23, 24]. JA signaling is negatively regulated by JAZs, which inhibit the expression of JA responsive genes by binding to the transcription factors such as MYC2 [25, 26]. In response to environmental stimulus, JA promotes the interaction between JAZs and the SCF^COI1^ ubiquitin ligase to trigger JAZ degradation via the ubiquitin/26S proteasome pathway [27]. The rapid activation of JA biosynthesis is stimulated by wound, a common response in plant development amid biotic and abiotic stresses such as insect attack, pathogen infection mechanical damage [23, 24]. In *Arabidopsis*, anthocyanin biosynthesis is induced by wound [28], and anthocyanin accumulation was regulated by JA signaling via the degradation of JAZ proteins to release bHLH and MYB TFs in the MBW complex [29]. In cotton, *F3H* and its downstream genes in proanthocyanidins (PAs) biosynthesis were significantly induced by *V. dahliae* infection and wound [30]. In apple tree, MdWRKY40 functions as a key modulator in the wounding-induced anthocyanin biosynthesis [31].

To date, many anthocyanin associated R2R3-MYB TFs have been isolated from different plants [16, 32, 33]. However, their regulatory mechanisms in wound induced anthocyanin biosynthesis are still largely unknown. Previously, we demonstrated that PdMYB118 regulated the biosynthesis of anthocyanin in poplar [34]. In this work, we report that PdMYB118 functioned in anthocyanin biosynthesis by interacting with PdTT8 to activate the expression of *ABGs* in poplar. Wound treatment induced JA accumulation, triggered the degradation of PtrJAZ1, and released its repression on the transcriptional activation activity of PdMYB/PdTT8 complex by directly interacting with PdTT8, and then activated the expression of *ABGs* for anthocyanin biosynthesis.

## Results

### PdMYB118 is involved in wound induced anthocyanin biosynthesis in poplar

**Fig. 1:**
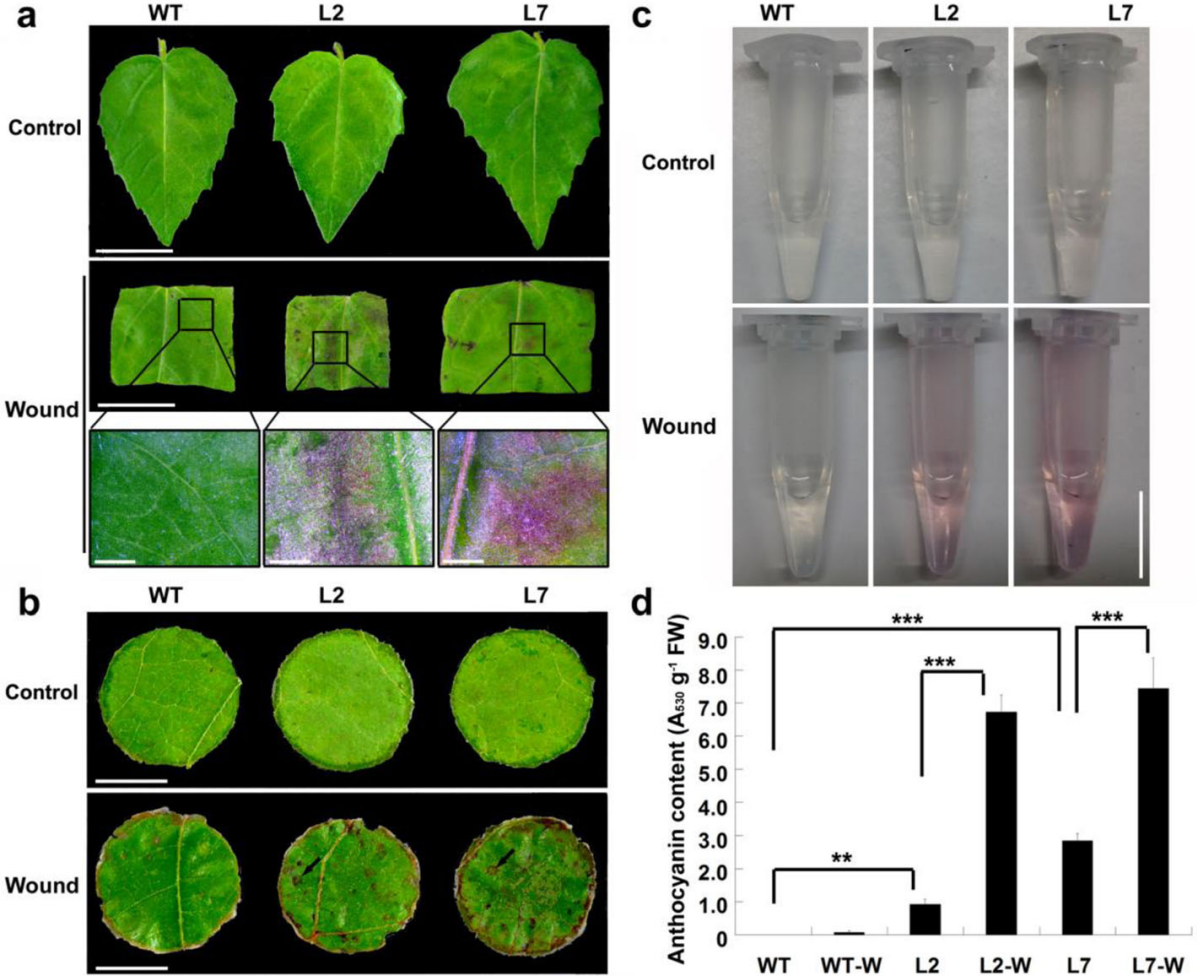
Wound induced accumulation of anthocyanins in the leaves of transgenic plants overexpressing *PdMYB118*. **a** Wound prompts anthocyanin biosynthesis in the leaves of transgenic plants. The leaves of WT and transgenic plantlets cultured on MS medium were cut into pieces and transferred onto MS medium for 2 days. Images in the third row were the higher magnification of the framed area of the images in the secondary row. Scale bars for the images in the first and secondary rows stand for 1 cm and in the third row stand for 1 mm. **b** Accumulated anthocyanin biosynthesis in the wounded leaves of transgenic plants grown in greenhouse. Matured leaves were cut into leaf discs and transferred onto MS medium for 2 days. The black arrows indicate the red speckles. Scale bar = 0.5 cm. **c** Anthocyanin extracted from the wounded leaf discs in (b). Scale bar = 0.5 cm. **d** Relative content of anthocyanins extracted from the leaves in (b). Control, before wound induction; Wound, after wound induction on MS medium for 2 days; WT, leaves of wild type Shanxin Yang plants; L2 and L7, leaves of transgenic plants overexpressing *PdMYB118*; WT-W, wounded leaves of wild type plant; L2-W and L7-W, wounded leaves of transgenic plants (Lines L2 and L7). Values are means and standard deviations of three biological replicates (*n* = 3). ** and ***, significant differences in comparison to WT, L2 and L7 at *P* < 0.01 and *P* < 0.001, respectively (Student’s t-test)

To explore the possible functions of *PdMYB118* in anthocyanin biosynthesis in poplar, we previously generated transgenic Shanxin Yang plants [34]. Overexpression of *PdMYB118* led to red leafed phenotype in adult transgenic plants grown in both green house and field, whereas young tissue culture plantlets showed green leaves as did the wide type (WT) (Additional file 1: Fig. S1a). When young plantlets were sub-cultured onto new MS medium, their leaves turned red and produced more anthocyanins after being cut off from their mother plants (Additional file 1: Fig. S1b-d). Therefore, wound may have prompted the biosynthesis of anthocyanin in transgenic plants. To confirm this speculation, wound induction was applied to both young leaves of tissue cultured transgenic plantlets and mature leaves of greenhouse grown transgenic plants. As we have hypothesized, both WT and transgenic leaves from tissue cultured plantlets were green, but transgenic leaf discs formed more red speckles 2 days after wound induction (Fig. 1a). Similar results were also observed in the mature leaf discs of greenhouse grown transgenic plants (Fig. 1b). Transgenic leaf discs produced more anthocyanins than did the WT control (Fig. 1c, d). These results indicate that PdMYB118 is involved in wound induced anthocyanin biosynthesis in poplar.

### Wound induces the expression of *ABGs*

**Fig. 2:**
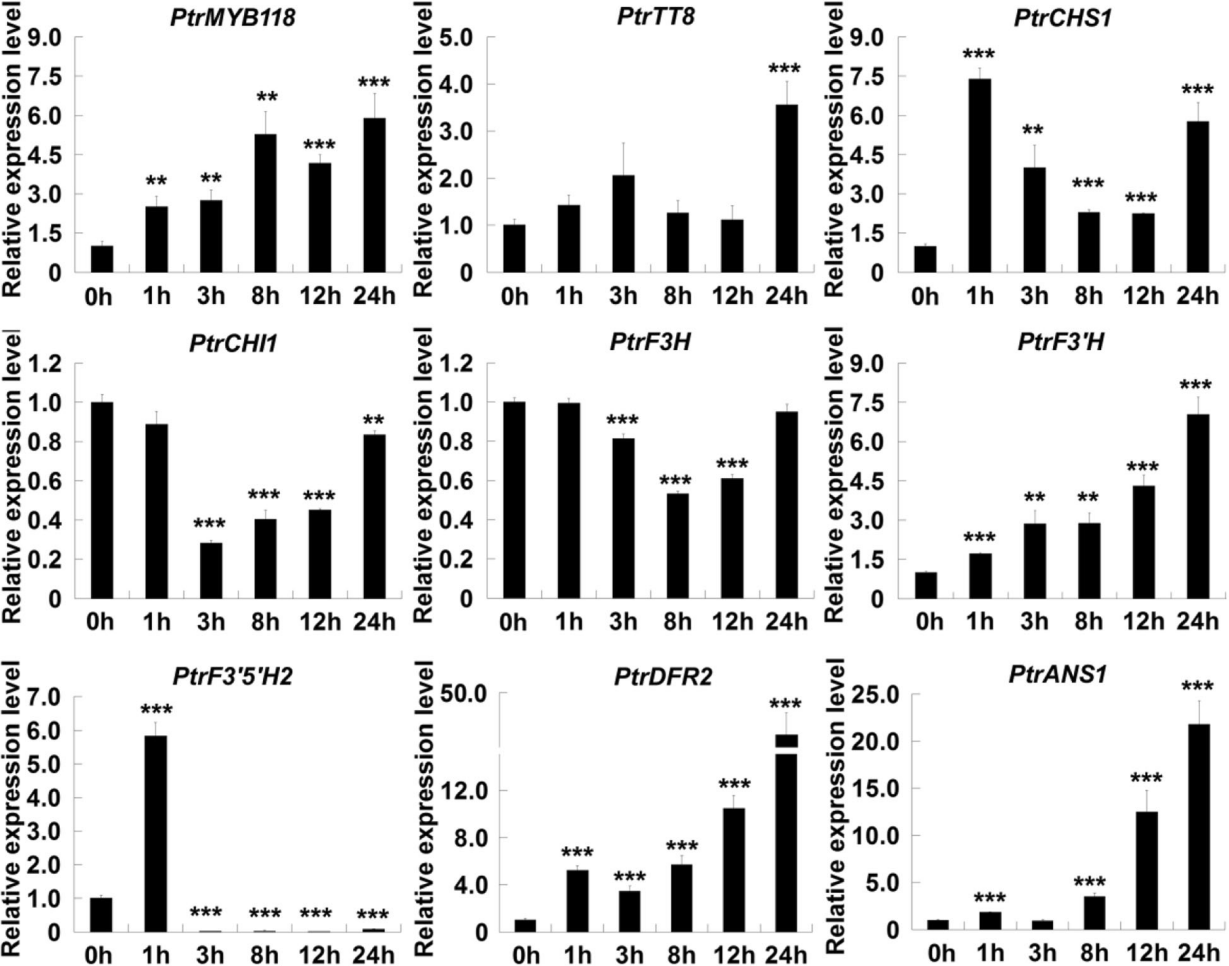
qRT-PCR analyses of *PdMYB118* and *ABGs* in the wounded leaves of wild type poplar plants. The mature leaves of poplar plants grown in greenhouse were treated using leaf-disc method for various time intervals for RNA extraction. Gene expression in the untreated leaves (0 h) was set to 1. *PtrMYB118* and *PtrTT8* are respectively the homology of PdMYB118 and PdTT8, and *PtrCHS1*, *PtrCHI1*, *PtrF3H*, *PtrF3’H*, *PtrF3’5’H*, *PtrDFR2* and *PtrANS1* are the *ABG* genes respectively encoding chalcone synthase, chalcone isomerase, flavanone 3-hydroxylase, flavanone 3′-hydroxylase, flavonoid 3′5’-hydroxylase, dihydroflavonol 4-reductase and anthocyanidin synthase in *Populus*. Three independent replicates of measurements were performed for each time point, and the values are means and standard deviations (*n* = 3). ** and ***, significant differences at *P* < 0.01 and *P* < 0.001 (Student’s t-test)

Based on the observation that wound promoted anthocyanin accumulation in *PdMYB118* overexpressing transgenic plants, we postulated that wound signaling may also induce the expression of *ABGs* in wild type poplar. We found that *PdMYB118* transcripts were gradually increased in the leaf discs during wound induction, and reached to the highest transcription level at 24 h (Fig. 2). Some *ABGs*, including *PtrF3’H*, *PtrDFR2* and *PtrANS1*, showed similar expression pattern as *PdMYB118*. A responsive expression of *PtrCHS1* to wound induction was also observed: it increased at 1 h, then gradually reduced within 12 h and restored to the high expression level at 24 h. *PdTT8* was slight induced in wounded leaves within 3 h, and obviously up-regulated at 24 h. *PtrCHI1* and *PtrF3H* were down-regulated within 3 to 12 h after wound induction and then reached to the normal expression level. The transcripts of *PtrF3’5’H2* reached a peak level within 1 h after wound induction and rapidly decreased to lower levels at later time points. From these data, we identified four wound-inducible *ABGs*: *PtrCHS1*, *PtrF3’H*, *PtrDFR2* and *PtrANS1*.

### The transcriptional activation activity of PdMYB118 is enhanced by a bHLH TF PdTT8

**Fig. 3:**
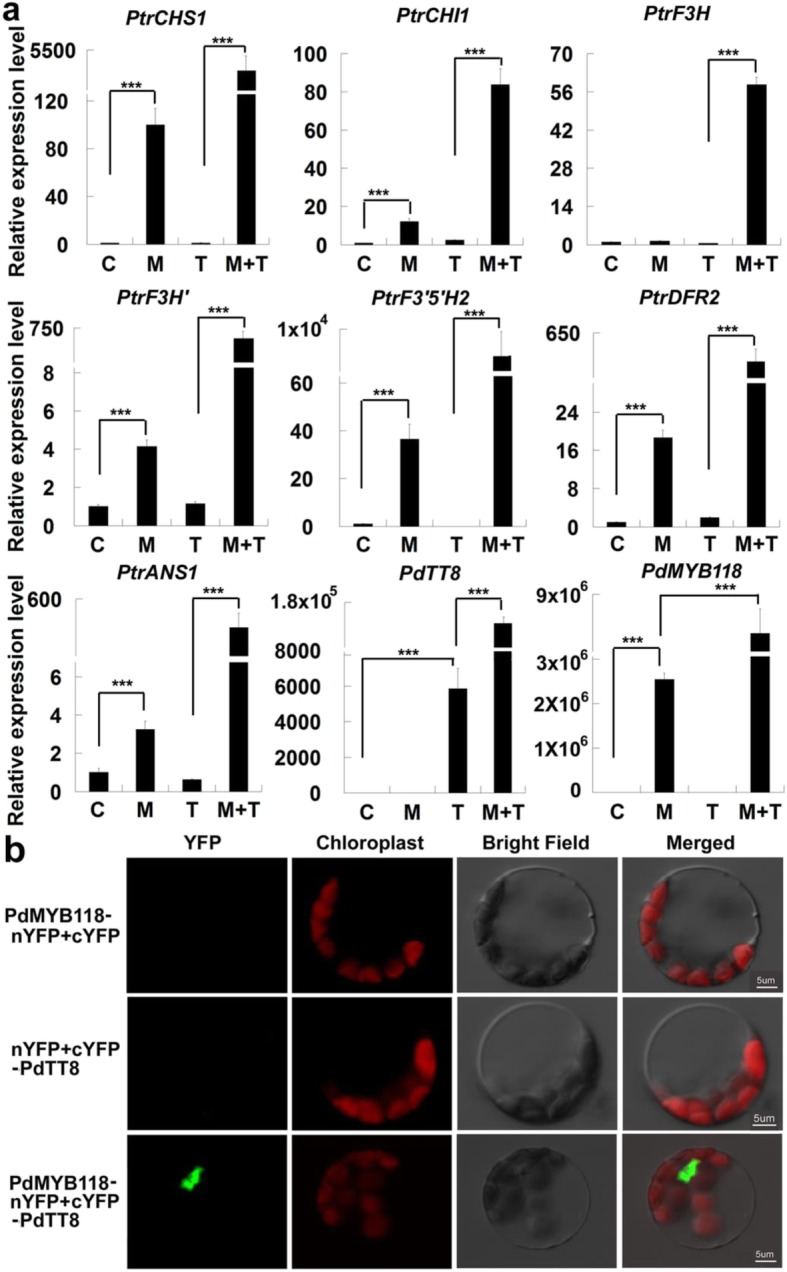
PdTT8 enhances the transcriptional activation activity of PdMYB118. **a** Transient expression of *PdMYB118* or *PdTT8* in poplar mesophyll protoplasts. The constructs of pGreenII62-SK-*PdMYB118*, pGreenII62-SK-*PdTT8* or pGreenII62-SK-*PdMYB118* + pGreenII62-SK-*PdTT8* were transfected into the poplar leaf protoplasts, respectively. The empty vector was used as a negative control. RNA was extracted from the transfected protoplasts for qRT-PCR analyses of *ABGs*, *PdTT8* and *PdMYB118*. *PtrCHS1*, *PtrCHI1*, *PtrF3H*, *PtrF3’H*, *PtrF3’5’H*, *PtrDFR2* and *PtrANS1* are the *ABG* genes respectively encoding chalcone synthase, chalcone isomerase, flavanone 3-hydroxylase, flavanone 3′-hydroxylase, flavonoid 3′5’-hydroxylase, dihydroflavonol 4-reductase and anthocyanidin synthase in *Populus*. C, protoplasts transfected with pGreenII62-SK (control); M, protoplasts transfected with pGreenII62-SK-*PdMYB118*; T, protoplasts transfected with pGreenII62-SK-*PdTT8*; M + T; protoplasts transfected with pGreenII62-SK-*PdMYB118* and pGreenII62-SK-*PdTT8*. **b** BiFC assays to detect the interaction of PdTT8 and PdMYB118. PdMYB118 or PdTT8 was respectively fused with N-terminal and C-terminal fragments of YFP. Construct pairs indicated on the left were co-expressed in the leaf protoplasts of WT poplar plants. Gene expression level in the control sample was set to 1. Values are means and standard deviations of three biological replicates (*n* = 3). ***, significant difference in comparison to C and T at *P* < 0.001, respectively (Student’s t-test)

To clarify the exact function of *PdMYB118* in wound induced anthocyanin biosynthesis, we first transiently expressed it in the leaf protoplasts of wild type Shanxin Yang plants. We found that transient expression of *PdMYB118* enhanced the transcription of most *ABGs*, including the wound-/JA-inducibe *ABGs*: *PtrCHS1*, *PtrF3’H*, *PtrDFR2* and *PtrANS1* (Fig. 3a). In plants, anthocyanin-related MYB factors interact closely with bHLH TFs to control anthocyanin biosynthesis [15]. We cloned a poplar bHLH TF PdTT8, the homolog of MdbHLH3 in apple and TT8 in *Arabidopsis*, which interacted with different MYB TFs, and investigated its transcription activity. PdTT8 alone did not regulate the expressions of *ABGs*, but could significantly enhance the transcriptional activation activity of PdMYB118. When *PdTT8* was transiently co-expressed with *PdMYB118* in the protoplasts, the transcription levels of all *ABGs* were remarkably higher than those expressing *PdMYB118* alone (Fig. 3a). These results imply that PdTT8 could be an efficient enhancer of PdMYB118 to regulate the expression of *ABGs*.

The enhanced transcriptional activation function of PdMYB118 by PdTT8 suggested a possible interaction of these two proteins. Therefore, we performed BiFC assays to confirm this possibility. The N- and C-terminal fragment of yellow fluorescent protein (nYFP and cYFP) was fused with PdMYB118 and PdTT8, respectively. As expected, when PdMYB118-nYFP and cYFP-PdTT8 were co-transfected into poplar leaf mesophyll protoplasts, the nuclei of protoplasts showed strong YFP fluorescence; whereas no signal was detected in the control (Fig. 3b). These results demonstrate that PdTT8 could directly interact with PdMYB118 to enhance its transcriptional activation activity.

### Wound prompts JA biosynthesis and JAZ1 degradation

**Fig. 4:**
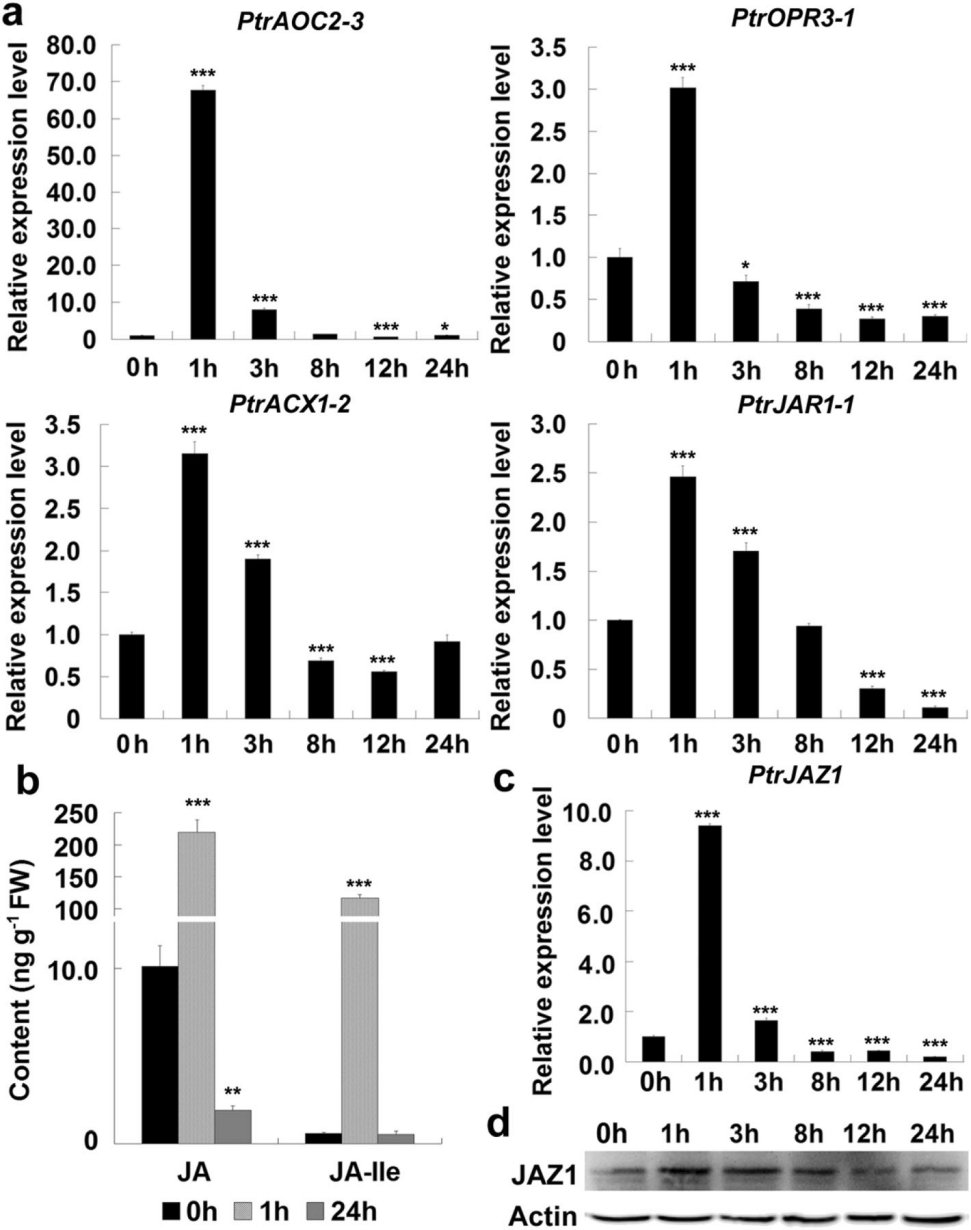
JA biosynthesis gene expression, JA content and JAZ1 degradation analyses. The mature leaves of poplar plants grown in greenhouse were treated using leaf-disc method for various time intervals and then used for RNA extraction. **a** qRT-PCR analyses of JA biosynthesis genes in the wounded leaves. *PtrAOC2–3*, a putative allene oxide cyclase gene in *Populus*; *PtrOPR3–1*, a putative OPDA reductase 3 gene in *Populus*; *PtrACX1–2*, a putative acyl-CoA oxidase gene in *Populus*; *PtrJAR1–1*, a putative JASMONATE RESISTANT gene (a JA-Ile biosynthesis gene) in *Populus*. **b** JA and JA-Ile content in the wounded leaves of poplar plants grown in greenhouse. The leaves treated for various time intervals were used to be tested. **c** qRT-PCR analyses of *PtrJAZ1* expression in the wounded leaves. **d** Western blotting analyses of JAZ1 protein in the wounded leaves. Total proteins were extracted from the leaf-discs treated for various time intervals. An amount of 30 μg proteins were separated by 10% SDS-PAGE and hybridized with *Arabidopsis* JAZ1 antibodies (JAZ1) or plant actin antibodies (Actin), respectively. Each gene expression in the untreated leaves (0 h) was set to 1. Three independent replicates of measurements were performed for each time point, and the values are means and standard deviations (*n* = 3). * and ***, significant differences at *P* < 0.05 and *P* < 0.001 (Student’s t-test). The original uncropped blot image was shown in additional file 6: Fig. S6

JA biosynthesis is catalyzed by a series of biosynthetic enzymes step by step [26]. It is widely considered that JA is rapidly synthesized in plant leaves suffering wound stimulus [23, 24]. To detect the JA changes in response to wound, we examined the expressions of JA biosynthesis genes *PtrAOC2–3*, *PtrOPR3–1*, *PtrACX1–2* and *PtrJAR1–1*. All these genes were rapidly expressed to their peak levels within 1 h after wound induction and then reduced gradually as the treatment elongated (Fig. 4a). The responsive expression of JA biosynthesis genes may result in JA accumulation. We then measured JA and JA-Ile contents at two time points: 1 h (short time) and 24 h (long time) after mechanical damage. JA and JA-Ile contents were about 10 ng g^− 1^ and 0.58 ng g^− 1^ fresh weight tissues (FW) in undamaged leaves, respectively. However, after 1 h of wound induction, the contents of JA and JA-Ile increased by about 16 and 200 folds (Fig. 4b). After 24 h, JA and JA-Ile contents reduced to about 1.9 and 0.53 ng g^− 1^ FW. The changes in JA and JA-Ile contents in wounded poplar leaves were tightly correlated with the expression changes of JA biosynthesis genes.

It has been reported that *JAZ* genes are obviously induced by mechanical wounding [23]. We analyzed the expression of a poplar *JAZ* gene *PtrJAZ1* during wound induction, and found that *PtrJAZ1* transcripts were quickly up-regulated within 1 h after wound induction, rapidly decreased within 3 h, and dropped to the lowest level after 24 h (Fig. 4c). The change in PtrJAZ1 protein content in response to wound induction was then tested by Western blotting. Consistent with its gene expression variation in wounded leaves, PtrJAZ1 protein accumulation reached to a higher level within 3 h, began to decline after 8 h, and reduced to a lower level after 12 h (Fig. 4d). These results indicate that wound induced anthocyanin biosynthesis in poplar may be mediated by JA signaling.

### JA regulates the expressions of *ABGs* in poplar

**Fig. 5:**
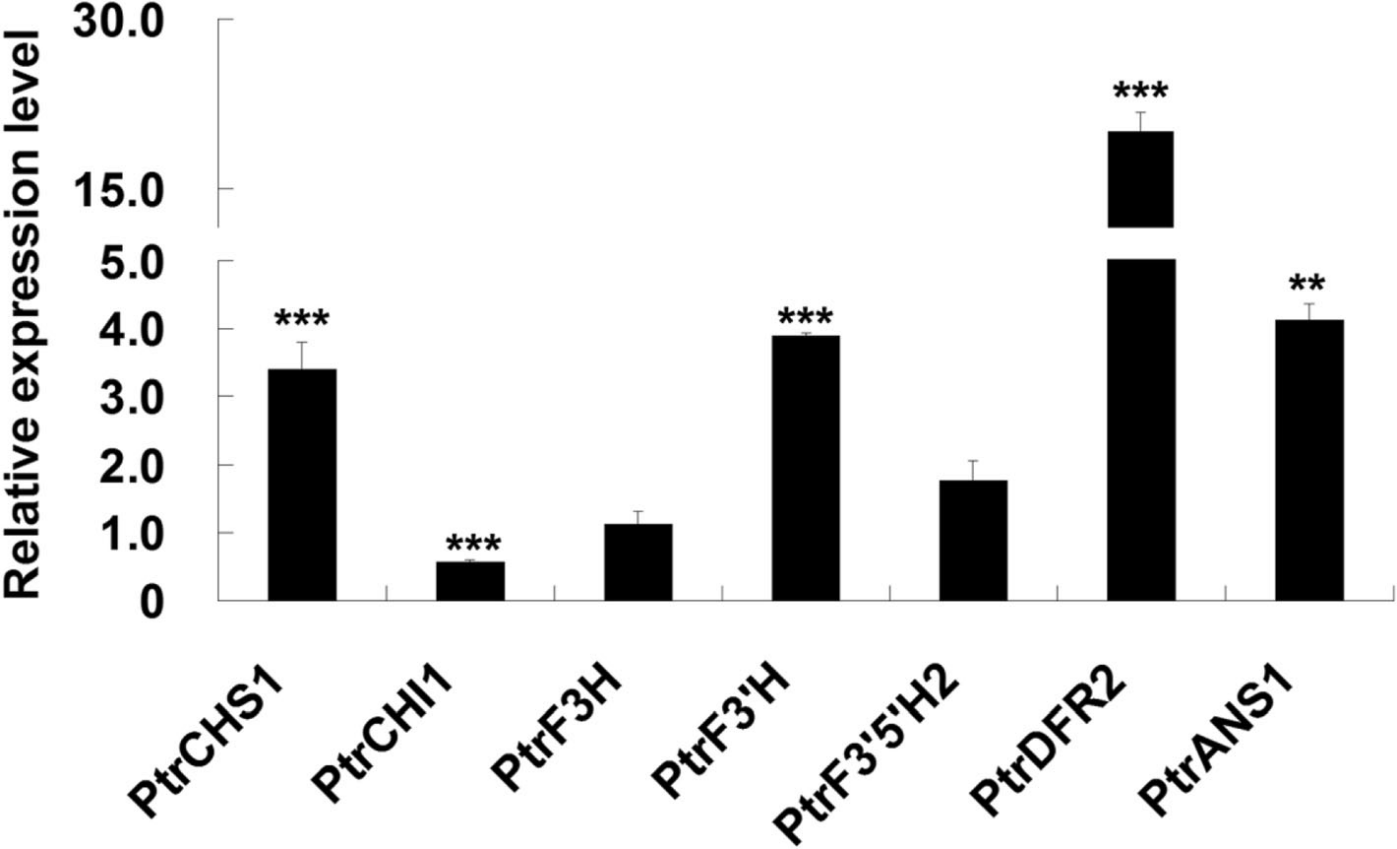
qRT-PCR analyses of *ABGs* in MeJA treated leaves. *PtrCHS1*, *PtrCHI1*, *PtrF3H*, *PtrF3’H*, *PtrF3’5’H*, *PtrDFR2* and *PtrANS1* are the *ABG* genes respectively encoding chalcone synthase, chalcone isomerase, flavanone 3-hydroxylase, flavanone 3′-hydroxylase, flavonoid 3′5’-hydroxylase, dihydroflavonol 4-reductase and anthocyanidin synthase in *Populus*. Gene expression in the leaves sprayed with water (control) was set to 1. Values are means and standard deviations of three biological replicates (*n* = 3). ** and ***, significant differences at *P* < 0.01 and *P* < 0.001 (Student’s t-test)

**Fig. 6:**
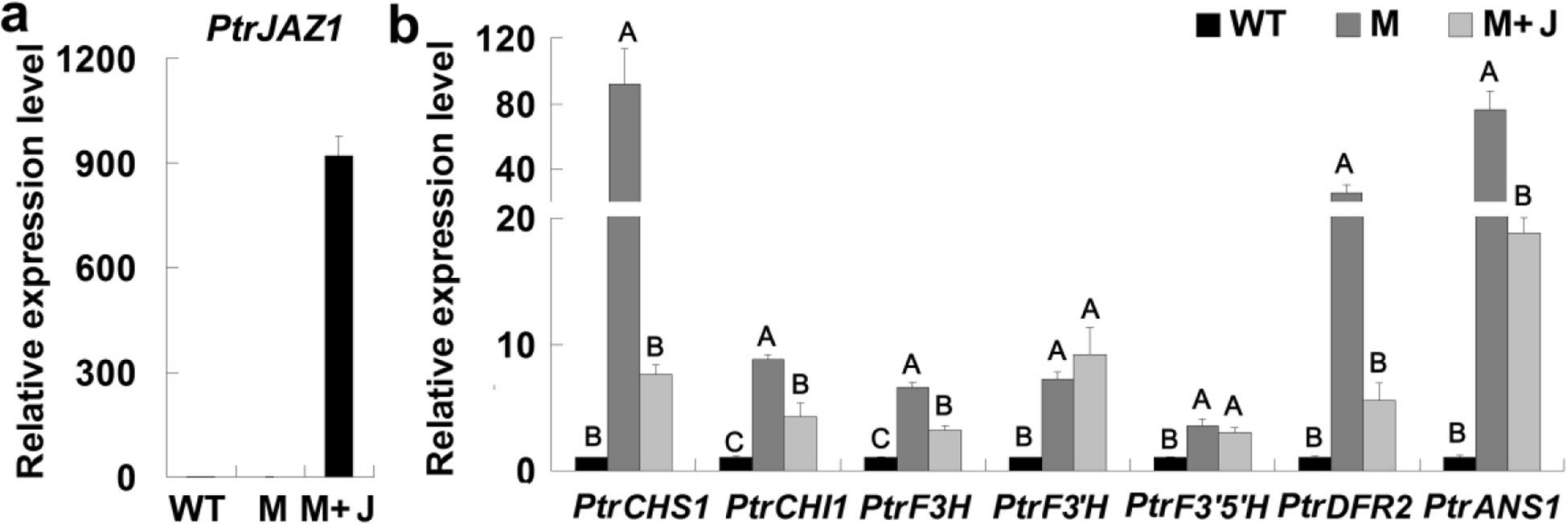
Expression levels of *ABGs* in transgenic protoplasts transiently expressing *PtrJAZ1*. Protoplasts isolated from the leaves of WT and transgenic plants overexpressing *PdMYB118* were used for the analyses. **a** Expression level of *PtrJAZ1*. **b** Expression levels of the *ABG* genes *PtrCHS1*, *PtrCHI1*, *PtrF3H*, *PtrF3’H*, *PtrF3’5’H*, *PtrDFR2* and *PtrANS1* respectively encoding chalcone synthase, chalcone isomerase, flavanone 3-hydroxylase, flavanone 3′-hydroxylase, flavonoid 3′5’-hydroxylase, dihydroflavonol 4-reductase and anthocyanidin synthase in *Populus*. Gene expression level in the WT was set to 1. WT, wild type protoplasts transfected with pGreenII62-SK; M, transgenic protoplasts transfected with pGreenII62-SK; M + J, transgenic protoplasts transfected with pGreenII62-SK-*PtrJAZ1*. Values (*n* = 3) are mean ± SD and different letters on the histograms indicate that the values differ significantly (one-way ANOVA; *P* < 0.01)

We further examined whether JA can regulate the expression of *ABGs*. Poplar leaves were sprayed with MeJA solution for qRT-PCR analyses. As expected, the expression levels of wound inducible *ABGs* (*PtrCHS1*, *PtrF3’H*, *PtrDFR2* and *PtrANS1*) were up-regulated more than 3 to 20 folds by MeJA treatment (Fig. 5). JA signaling is regulated by JAZ proteins, which inhibit the expression of JA response genes by binding to other transcription factor [35]. We transiently expressed *PtrJAZ1* in the leaf protoplasts of *PdMYB118* transgenic plants. The up-regulated expression of most *ABGs* by *PdMYB118* was inhibited by the expression of *PtrJAZ1* (Fig. 6a, b). These results indicate that JA induced expression of *ABGs* in poplar is negatively regulated by PtrJAZ1.

### PtrJAZ1 represses the transcriptional activation activity of PdMYB118/PdTT8 complex by binding to PdTT8

**Fig. 7:**
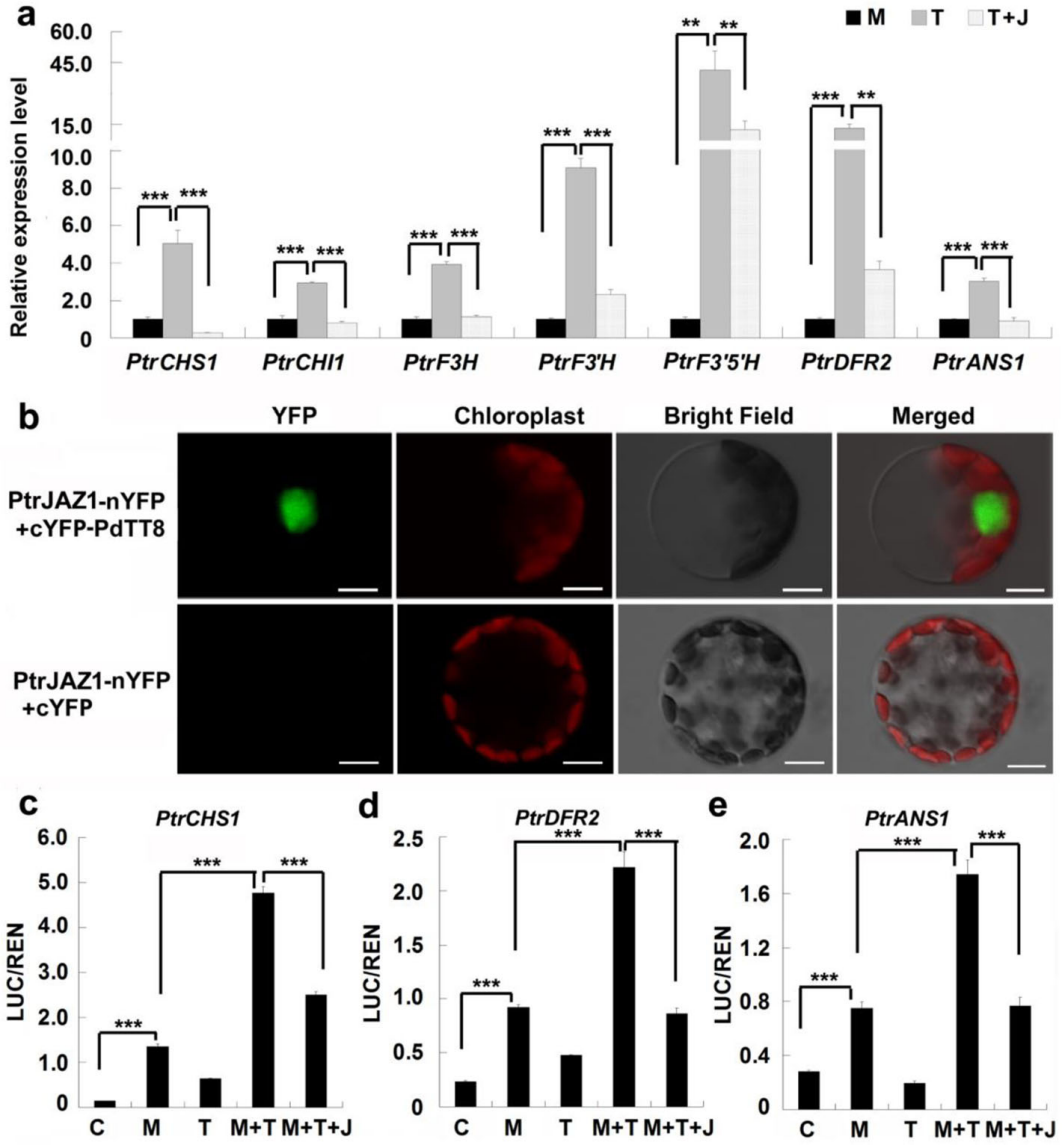
PtrJAZ1 binds to PdTT8 to inhibit the transcriptional activation activity of the PdMYB118/PdTT8 complex. *PtrCHS1*, *PtrCHI1*, *PtrF3H*, *PtrF3’H*, *PtrF3’5’H*, *PtrDFR2* and *PtrANS1* are the *ABG* genes respectively encoding chalcone synthase, chalcone isomerase, flavanone 3-hydroxylase, flavanone 3′-hydroxylase, flavonoid 3′5’-hydroxylase, dihydroflavonol 4-reductase and anthocyanidin synthase in *Populus*. **a** PtrJAZ1 represses the transcriptional activation activity of the PdMYB118/PdTT8 complex. Protoplasts isolated from the leaves of transgenic plants overexpressing *PdMYB118* were transformed with pGreenII62-SK-*PdTT8* alone, or co-transformed with pGreenII62-SK-*PdTT8* and pGreenII62-SK-*PtrJAZ1*. qRT-PCR analysis of *ABGs* in the transfected protoplasts was performed. The empty vector was used as a negative control and the gene expression level in the control was set to 1. Values are means and standard deviations of three biological replicates (*n* = 3). ** and ***, significant differences at *P* < 0.01 and *P* < 0.001 (Student’s t-test). M, transgenic protoplasts transfected with pGreenII62-SK; T, transgenic protoplasts transfected with pGreenII62-SK-*PdTT8*; T + J, transgenic protoplasts co-transfected with pGreenII62-SK-*PdTT8* and pGreenII62-SK-*PtrJAZ1*. **b** BiFC assays to test the interaction of PtrJAZ1 with PdTT8. PtrJAZ1 was fused with the N-terminal fragment of YFP, and PdTT8 was linked to the C-terminal fragment of YFP, respectively. Construct pairs indicated on the left were co-expressed in poplar leaf protoplasts. Scale bar = 10 μm. **c-e** Transient transcription dual-luciferase assays to show that PtrJAZ1 inhibits the transcriptional activation activity of the MYB118/TT8 complex to the promoters of *PtrCHS1* (c), *PtrDFR2* (d) and *PtrANS1* (e). The effectors (pGreenII62-SK-*PdMYB118*, pGreenII62-SK-*PdTT8*, pGreenII62-SK-*PdMYB118* + pGreenII62-SK-*PdTT8 or* pGreenII62-SK-*PdMYB118* + pGreenII62-SK-*PdTT8 +* pGreenII62-SK-*PtrJAZ1*) and reporters (*35S*::*REN*-*ProPtrCHS1*::*LUC*, *35S*::*REN*-*ProPtrDFR2*::*LUC* or *35S*::*REN*-*ProPtrANS1*::*LUC*) were co-expressed in poplar protoplasts. The expression level of REN was used as an internal control. The LUC/REN ratio represents relative activity of the *PtrCHS1*, *PtrDFR2* and *PtrANS1* promoters. Error bars represent the SDs from three biological replicates. C, protoplasts transfected with pGreenII62-SK (control); M, protoplasts transfected with pGreenII62-SK-*PdMYB118*; T, protoplasts transfected with pGreenII62-SK-*PdTT8*; M + T, protoplasts transfected with pGreenII62-SK-*PdMYB118* and pGreenII62-SK-*PdTT8*; M + T + J, protoplasts transfected with pGreenII62-SK-*PdMYB118*, pGreenII62-SK-*PdTT8* and pGreenII62-SK-*PtrJAZ1*. Values are means and standard deviations of three biological replicates (*n* = 3). ***, significant differences at *P* < 0.001 (Student’s t-test)

The suppressed transcriptional activation activity of PdMYB118 by PtrJAZ1 could be regulated by PdTT8. Therefore, we transiently expressed *PdTT8* and *PtrJAZ1* alone or together in the leaf protoplasts of *PdMYB118* transgenic plants (Additional file 2: Fig. S2a, b). We observed that transient expression of *PdTT8* in the leaf protoplasts of *PdMYB118* transgenic plants further increased the expression levels of *ABGs* (Fig. 7a). However, when *PtrJAZ1* and *PdTT8* were co-expressed in the leaf protoplasts of *PdMYB118* transgenic plants, the expression levels of *ABGs* were repressed to the levels of control samples (Fig. 7a). These results suggest that PtrJAZ1 suppressed the transcription activity of PdMYB118/PdTT8 complex. Then, we performed BiFC assays to examine whether PtrJAZ1 interacts with PdMYB118 or PdTT8. PtrJAZ1-nYFP was transiently co-expressed with cYFP-PdTT8 or cYFP-PdMYB118 in the leaf mesophyll protoplasts of wide type poplar. Co-expression of PtrJAZ1-nYFP with cYFP-PdTT8 produced strong YFP fluorescence in the nuclei of protoplasts, whereas no fluorescence signal was detected in the protoplasts co-expressing PtrJAZ1-nYFP and cYFP-PdMYB118 (Fig. 7b; Additional file 3: Fig. S3). This result is consistent with the finding in apple that MdJAZs interact with MdbHLH3 (the analogue of TT8) but not with the PA biosynthesis associated MdMYB9/11 [36].

We further confirmed the inhibition of PtrJAZ1 on the transcriptional activation activity of PdMYB118/PdTT8 complex by dual-luciferase assays. Three wound inducible *ABGs* (*PtrCHS1*, *PtrDFR2* and *PtrANS1*) were selected. The transcriptional activation activity of PdMYB118 on the promoters of *PtrCHS1*, *PtrDFR2* and *PtrANS1* was higher than that of PdTT8, showing a higher ratio of LUC/REN (Fig. 7c-e). The highest ratio values were observed in the protoplasts co-expressing PdMYB118 and PdTT8. And the transcription activity of PdMYB118/PdTT8 complex decreased obviously when PtrJAZ1 was co-transformed with PdMYB118 and PdTT8 (Fig. 7c-e). These results suggest that by binding to PdTT8, PtrJAZ1 could restrain the transcriptional activation activity of PdMYB118/PdTT8 complex.

### Degradation of JAZ1 proteins promotes anthocyanin biosynthesis in the wounded leaves of transgenic plants

**Fig. 8:**
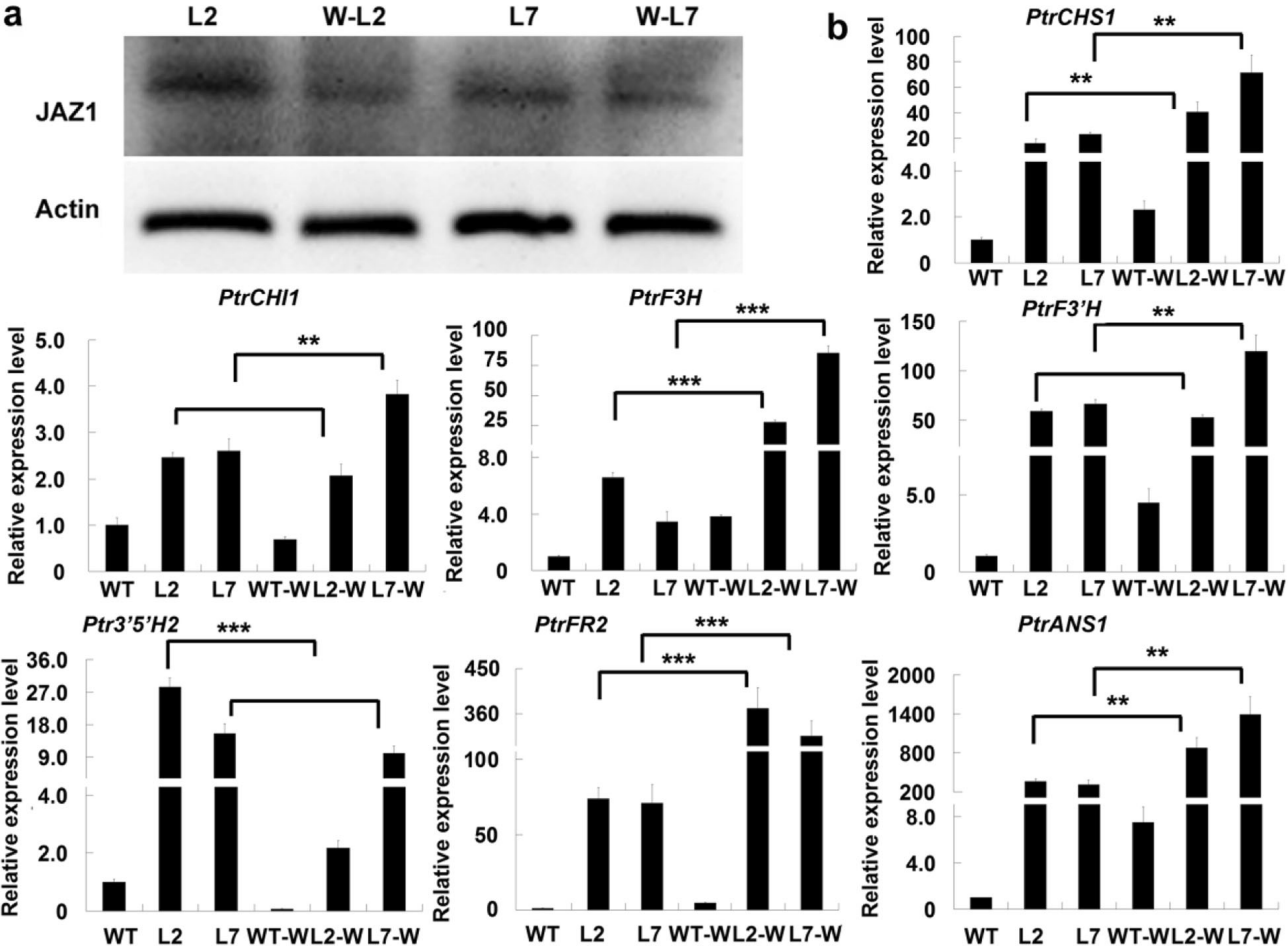
JAZ1 protein content and *ABG* expressions in the wounded leaves of transgenic poplar plants. *PtrCHS1*, *PtrCHI1*, *PtrF3H*, *PtrF3’H*, *PtrF3’5’H*, *PtrDFR2* and *PtrANS1* are the *ABG* genes respectively encoding chalcone synthase, chalcone isomerase, flavanone 3-hydroxylase, flavanone 3′-hydroxylase, flavonoid 3′5’-hydroxylase, dihydroflavonol 4-reductase and anthocyanidin synthase in *Populus*. **a** Western blotting analysis of JAZ1 contents. Total proteins were extracted from the untreated or treated leaves of *PdMYB118* overexpressing plants. JAZ1 antibodies (JAZ1) and plant actin antibodies (Actin) were used for the hybridization. The original uncropped blot image was shown in additional file 7: Fig. S7. **b** qRT-PCR analyses of *ABGs* in the untreated or treated leaves of wild type and *PdMYB118* overexpressing plants. The leaves were mechanically wounded by leaf-disc method for 2 days. Then RNA was extracted for qRT-PCR analyses. Gene expression level in wide type leaves was set to 1. Values are means and standard deviations of three biological replicates (*n* = 3). ** and ***, significant differences at *P* < 0.01 and *P* < 0.001 (Student’s t-test). WT, leaves of wide type poplar plant; L2 and L7, leaves of transgenic plants overexpressing *PdMYB118*; WT-W, wounded leaves of wide type poplar; L2-W and L7-W, wounded leaves of transgenic plants

Based on the observations that expression of *ABGs* was inhibited by PtrJAZ1 and JAZ1 proteins were degraded upon wound induction, we speculated that the increased anthocyanin biosynthesis in the wounded transgenic leaves may be a result of JAZ1 degradation. By Western blotting analysis using *Arabidopsis* JAZ1 antibodies, we found that JAZ1 accumulation in the wounded leaves of transgenic plants were distinctly lower than that in the no-treated control leaves (Fig. 8a). Therefore, the decreased JAZ1 protein level may have released the transcriptional activation activity of the MYB118/TT8 complex and increased the expression of wound-inducible *ABGs*. We further analyzed the expression of *ABGs* in the leaves of transgenic plants overexpressing *PdMYB118*. In the untreated leaves of transgenic plants (lines L2 and L7), wound−/JA-inducible *ABG*s (*PtrCHS1*, *PtrDFR2*, *PtrF3H* and *PtrANS1*) were up-regulated in transgenic plants. Upon wound induction, their expression levels increased in the leaves of both WT and transgenic plants, with a more significant increase in the wounded leaves of transgenic plants (Fig. 8b). Similarly, the expression level of *PdMYB118* increased in the wounded leaves of both WT and transgenic plants, but the increase was significantly higher in transgenic plants (Additional file 4: Fig. S4a). However, the expression level of *PdTT8* was only slightly changed in the leaves of both WT and transgenic leaves (Additional file 4: Fig. S4b). These results indicate that the increased anthocyanin accumulation in the wounded leaves of transgenic plants could be due to the enhanced expression of *ABGs* resulted from the wound-induced degradation of JAZ1 proteins.

## Discussion

Although wound induced anthocyanin accumulation has been widely observed in plants, the molecular mechanism that how wound regulates anthocyanin biosynthesis in poplar still remains unknown [28, 37]. Recently, we reported that *PdMYB118* regulated the biosynthesis of anthocyanin in poplar [34]*.* Overexpression of *PdMYB118* obviously activated the expression of anthocyanin biosynthesis genes but not the two proanthocyanin synthesis genes, leading to red leafed phenotype in transgenic plants. Interestingly, during the process of shoot propagation, we observed that the leaves of tissue cultured plantlets or green house grown young transgenic plants remained in green or less red color, but once the shoots were cut off, the leaves of new cut shoots turn into red color (Additional file 1: Fig. S1a-d). The stability of this phenomenon (wound-induced anthocyanin biosynthesis) was further confirmed using green leaves of both tissue cultured plantlets and greenhouse grown plants (Fig. 1a-d).

Previous studies have showed that JA regulates WD-repeat/bHLH/MYB (WBM) complex-mediated anthocyanin accumulation via the interaction of JAZ proteins with bHLH and MYB transcription factors [29]. PA-specific R2R3-MYB factors like MYB134 in poplar and MdMYB9/11 in apple dramatically responded to wound treatment, whereas the anthocyanin biosynthesis R2R3-MYB factor MdMYB1 in apple did not [8, 36]. MYB/bHLH complex, such as PAP1/TT8 in *Arabidopsis*, AN4/AN1 in *Petunia hybrid*, VvMYBA1/VvMYC1 in grape, and MdMYB10/MdbHLH3 in apple, are also involved in anthocyanin synthesis [13, 38–40]. We found that expression of most *ABGs* was responsive to wound treatment (Fig. 2). The poplar bHLH TF PdTT8 physically interacted with PdMYB118 to form the PdMYB118/PdTT8 complex, and more efficiently activated the expressions of *ABGs* (Fig. 3a, b). Therefore, PdTT8 may function as an enhancer to promote the transcriptional activation activity of PdMYB118.

Wound can rapidly activate the biosynthesis of JA, which functions as an important plant defense signal in response to various stresses [23, 24]. In plants, the biosynthetic pathway of JA is initiated from the tri-unsaturated fatty acids α-linolenic acid (18:3) [26]. After two oxidation steps, α-linolenic acid is converted to an unstable allene oxide, which is subsequently catalyzed to 12-oxophytodienoic acid (OPDA) by an allene oxide cyclase (AOC) in chloroplasts. Then the second half of JA biosynthesis is finished in peroxisomes, where OPDA is reduced to OPC-8 by OPDA reductase 3 (OPR3) [41]. After β-oxidation, JA is transported to cytosol, where it is conjugated to amino acids by JA-amino acid synthetase (JAR1) to form jasmonoyl-isoleucine (JA-Ile), a major biologically active jasmonate among a growing number of jasmonate derivatives. We found that wound treatment rapidly up-regulated the expression of JA biosynthesis genes within 1 h (Fig. 4a), leading to the rapid output of JA and JA-Ile in the wounded leaves (Fig. 4b). These results indicate that JA biosynthesis responds rapidly to wound induction in poplar. Increased JA biosynthesis by wound treatment could activate the JA signaling. Indeed, similar to the expression pattern of JA biosynthesis gene, *PtrJAZ1* was also up-regulated within 1 h after the initiation of wound induction, and then gradually decreased (Fig. 4c). This result is consistent with previous reports that most *JAZ* genes were strongly induced within 1 h after wound induction and declined at later time points [23, 24]. JA signaling also promotes the degradation of JAZ transcriptional repressors through the activity of the E3 ubiquitin-ligase SCF^COI1^ JAZs [27]. We found that the content of poplar JAZ proteins was also obviously decreased after 8 h of wound treatment (Fig. 4d). Therefore, in poplar, wound induction could rapidly induce JA biosynthesis by activating its biosynthesis genes and promote the degradation of JAZ protein.

JA regulates several anthocyanin biosynthetic genes involved in anthocyanin accumulation [42, 43]. In poplar, MeJA treatment could induce the expression of some *ABGs* such as *PtrCHS1*, *PtrF3H’*, *PtrDFR2* and *PtrANS1* (Fig. 5). Interestingly, expressions of these *ABGs* were also induced by wound, indicating that wound-induced anthocyanin biosynthesis may be mediated by JA signaling. It has been reported that JA regulates WD-repeat/bHLH/MYB complex-mediated anthocyanin accumulation via the interaction of JAZ proteins with bHLH and MYB factors [29]. We found that PtrJAZ1 specifically bound to PdTT8 and attenuated the transcriptional activation activity of PdMYB118/PdTT8 complex on the promoter of JA/wound-inducible *ABGs* (Fig. 7a-e). JAZ regulated expression of *ABGs* in poplar is similar to that in *Arabidopsis* [29], but different from that in apple, where MdJAZ2 inhibits the recruitment of MdbHLH3 to the promoters of MdMYB9 and MdMYB11 which regulate the biosynthesis of anthocyanin and proanthocyanin [24]. Therefore, JA induced JAZ protein degradation could abolish the interaction of JAZ proteins with bHLH and MYB factors, and then activate the biosynthesis of anthocyanin [29]. In agreement with this speculation, when PtJAZ1 protein was degraded in the wounded leaves of transgenic plants, JA−/wound-inducible *ABGs* were highly induced to promote anthocyanin biosynthesis (Fig. 8a, b).

## Conclusions

Taken together, our study illustrates the biological function of PdMYB118 in wound-induced anthocyanin synthesis in poplar. Although more detailed studies are still needed to completely understand the mechanism of PdMYB118 in anthocyanin synthesis, our data presented here imply a possible model of wound-induced anthocyanin synthesis in poplar: upon wound induction, JA biosynthetic genes are rapidly activated for JA biosynthesis, subsequently triggered the degradation of PtrJAZ1; Then, the transcriptional activation activity of PdMYB118/PdTT8 complex is restored to promote the expression of anthocyanin biosynthesis genes; Finally, anthocyanins were biosynthesized, leading to red leafed phenotype in transgenic plants (Additional file 5: Fig. S5a, b).

## Methods

### Plant materials and growth conditions

Wild type Shanxin Yang (WT) and transgenic Shanxin Yang (*P. davidiana × P. bolleana*) overexpressing *PdMYB118* used in our previous report were propagated on MS medium, transferred to soil, and grown in greenhouse [34, 44]. Shanxin Yang was provided by Prof. Gui-Feng Liu (Northeast Forestry University, China). *Populus deltoids* and its red leaf mutant were purchased from Qingyuan HiTech Ltd. (Yantai, China). Plants were grown in greenhouse at 25 °C (day)/18 °C (night) in a 12 h light/12 h dark photoperiod.

### Wound treatments

For wound induction, the leaves of wild type and transgenic poplar grown on MS medium or in green house were cut into 1.5 cm dices and transferred onto MS medium. At various time points after wound induction, leaf discs were harvested, frozen in liquid nitrogen, and stored at − 80 °C for RNA, protein, JA and anthocyanin extraction.

### MeJA treatment

To analyze the induction of *PdMYB118* and *ABGs* by JA, 100 μM MeJA (Sigma, Shanghai, China) solution was sprayed onto the mature leaves of WT poplar plants grown in greenhouse. After 3 h, leaves were collected and used for RNA extraction. Leaves sprayed with water were used as a negative control.

### Quantitative real-time RT-PCR

Total RNA was extracted from leaves and leaf protoplasts with RNAiso Reagent (Takara, Shanghai, China) and qPCR was performed as described previously [45]. The relative expression of each target gene was normalized using *PtrEF1β*. Gene specific primers used in this study were listed in additional file 8: Table S1. Three independent replicates of measurements were performed for each sample.

### Anatomical observations

For histological observation of anthocyanin speckles, leaf dices of wild type and transgenic plants were examined with a light microscope after wound treatment for 48 h. Images were captured under the SMZ800 microscope.

### Anthocyanin content determination

After wound induction for 48 h, anthocyanin content in the leaf discs of WT and transgenic plants grown in greenhouse was measured as described previously [46]. To detect the quantity of anthocyanin, A_530_ per gram fresh weight (FW) was used. Three replicates were carried out for each measurement, and the variability was indicated with the standard deviation (SD).

### Western blotting

Western blotting analyses were performed as described previously [47]. Briefly, a total amount of 30 μg proteins were extracted from the wounded leaf discs at various time points and separated by 10% sodium dodecyl sulfate-polyacrylamide gel electrophoresis (SDS-PAGE). Then total protein was electrotransferred onto polyvinylidene difluoride membranes. Immunoblotting was performed using JAZ1 antibodies against the *Arabidopsis* JAZ1 protein (Agrisera, http://www.agrisera.com/), then incubated with the secondary antibody goat anti-rabbit IgG-horseradish peroxidase (HRP) (Abmart, China). To detect the Actin, the primary antibody (mouse monoclonal Actin antibody) and the secondary antibody (goat anti-mouse IgG-HRP) were used to perform the immune reaction (Abmart, Shanghai, China). After incubation in the chemiluminescence detection solution LumiGLO (KPL, USA), membranes were imaged with a chemiluminescence image system Tanon 5500 (Tanon, Shanghai, China). Proteins were quantified with a BCA Protein Assay kit (Thermo, Shanghai, China).

### JA content assays

For JA and JA-Ile content assays, the wounded leaves of wild type poplar plants at 0 h, 1 h and 24 h time points were ground into fine powder in liquid nitrogen, respectively. JA extraction was performed as described previously [48]. Three replicates were carried out for each assay, and the variability was indicated with the standard deviation (SD).

### Transient expression of TFs in poplar mesophyll protoplasts

To analyze the transcription function of PdMYB118, PdTT8 and PdMYB118/PdTT8 complex, CaMV 35S promoter driven transcriptional factor effectors were produced by inserting *PdMYB118* or *PdTT8* into pGreenII62-SK [49]. Leaf protoplasts were isolated from the leaves of Shanxin Yang as described previously [34]. The resultant constructs pGreenII62-SK-*PdMYB118*, pGreenII62-SK-*PdTT8* and pGreenII62-SK-*PdMYB118* + pGreenII62-SK-*PdTT8* were transferred into protoplasts, respectively. The empty vector was used as a negative control. After kept in dark for 16 h, the transfected protoplasts were collected for RNA extraction and qRT-PCR analyses of *ABGs* as described above.

To detect the effects of PdTT8 or PtrJAZ1 on *ABGs* expression in the leaves of transgenic plants overexpressing *PdMYB118*, effectors pGreenII62-SK-*PdTT8*, pGreenII62-SK-*PtrJAZ1* and pGreenII62-SK-*PdTT8* + pGreenII62-SK-*PtrJAZ1* were transformed into the mesophyll protoplasts of transgenic plants, respectively. The transfected protoplasts were used for qRT-PCR analyses of *ABGs*. Three replicates were carried out for each assay, and the variability was indicated with the standard deviation (SD).

### BiFC assays

Full-length coding sequences of *PtrJAZ1*, *PdMYB118* and *PdTT8* were individually cloned and subsequently recombined into YFP BiFC vectors so that they were fused with the N- or C-terminal of YFP (nYFP or cYFP) to generate pSAT4-nYFP-PdMYB118/PtrJAZ1 and pSAT4-cYFP-PdTT8/PdMYB118 plasmids. Primers used for gene clone are given in Supporting Information Table S1. To detect the interaction of PtrJAZ1 with PdTT8 or PdMYB118, and the interaction of PdMYB118 with PdTT8, the relative constructs were co-transfected into the mesophyll protoplasts of wild type plants. After incubated at 23 °C for 16 h, the protoplasts were analyzed using a confocal microscope at 514 nm wavelength (Zeiss LSM 510 META). Three replicates were carried out for each assay, and the variability was indicated with the standard deviation (SD).

### Transient transcription dual-luciferase assays

For dual-luciferase assays, the LUC reporter constructs were generated by cloning the promoter of *PtrCHS1*, *PtrDRF2* or *PtrANS1* into pGreenII0800-LUC [47]. The resultant pGreenII62-SK-PdMYB118, pGreenII62-SK-PdTT8 or pGreenII62-SK-PtrJAZ1 was used as effector construct as described above. To detect the inhibition of PtrJAZ1 to the transcription activity of the PdMYB118/PdTT8 complex, effectors PdMYB118, PdTT8, PdMYB118 + PdTT8 or PdMYB118 + PdTT8 + PtrJAZ1 were repetitively co-expressed with each reporter construct in poplar leaf protoplasts [45]. The LUC/REN ratio was used to represent the relative activity of the transcriptional factors. Three replicates were carried out for each assay, and the variability was indicated with the standard deviation (SD).

### Statistical analysis

All data were obtained from three biological replicates each. For statistical analyses, Student’s t-test (two-tailed) or ANOVA (one-way) was used to generate every *P* value. The variability was indicated with the standard deviation (SD). *, ** and *** indicate *p*-values < 0.05, <0.01 and < 0.001, respectively.

## Supplementary information

**Additional file 1: Figure S1.** Wound induced anthocyanin biosynthesis in the leaf of transgenic plants.

**Additional file 2: Figure S2.** BiFC assays to test the interaction of PtrJAZ1 with PdMYB118.

**Additional file 3: Figure S3.** Expression of *PtrJAZ1* and *PdTT8* in the protoplasts isolated from the leaves of transgenic plants overexpressing *PdMYB118.*

**Additional file 4: Figure S4.** qRT-PCR analyses of *PdMYB118* and *PdTT8* in the wounded leaves of WT and transgenic plants overexpressing *PdMYB118.*

**Additional file 5: Figure S5.** A proposed model of wound induced anthocyanin biosynthesis in poplar.

**Additional file 6: Figure S6.** Western blotting analyses of JAZ1 protein in the wounded leaves.

**Additional file 7: Figure S7.** Western blotting analyses of JAZ1 protein in the wounded leaves of transgenic poplar plants.

**Additional file 8: Table S1.** Primer sequences used in this study.

## Data Availability

All materials and data analyzed in this study are available from the corresponding author

## Abbreviations

TF: Transcription factors
ABG: Anthocyanin biosynthesis gene
MeJA: Methyl jasmonate
JAZ: JASMONATE ZIM-domain
CHS: Chalcone synthase
CHI: Chalcone isomerase
F3H: Flavanone 3-hydroxylase
F3’H: Flavanone 3′-hydroxylase
F3′5’H: Flavonoid 3’5’-hydroxylase
DFR: Dihydroflavonol 4-reductase
ANS: Anthocyanidin synthase
bHLH: Basic helix-loop-helix
WT: Wild type
