# Supplementary material for: MYB transcription factor PdMYB118 directly interacts with bHLH transcription factor PdTT8 to regulate wound-induced anthocyanin biosynthesis in poplar
Source: BMC Plant Biol. 2020 Apr 20;20:173. doi: 10.1186/s12870-020-02389-1 (PMC7168848; doi:10.1186/s12870-020-02389-1)
Supplement: Supplementary file 1 — Additional file 1: Figure S1. Wound induced anthocyanin biosynthesis in the leaf of transgenic plants. [file 12870_2020_2389_MOESM1_ESM.docx]

**
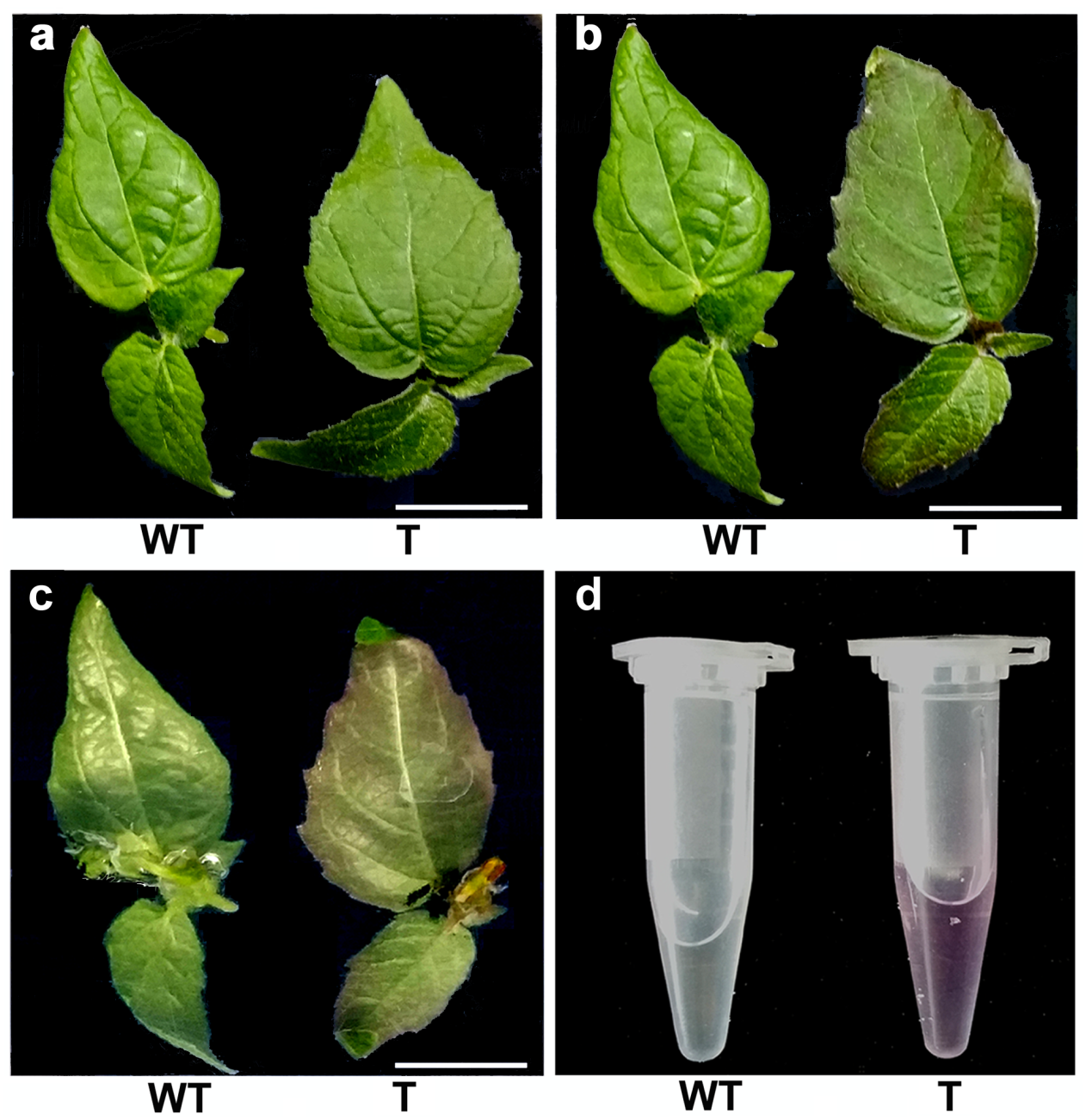
**

**Figure S1.** Wound induced anthocyanin biosynthesis in the leaf of transgenic plants. (a) Leaf colors of WT and transgenic poplar overexpressing *PdMYB118*. Initially, leaves from the shoots of wide type and transgenic plantlets were green. (b) Red color showed up on the adaxial side of transgenic plantlets after cultured on MS medium for 3 days. (c) Red color was more obvious on the abaxial side of transgenic plantlets. (d) Anthocyanins were extracted from the leaves described in (b) and (c). WT, wide type poplar; T, transgenic plants overexpressing *PdMYB118*. Scale bar = 1 cm.
