# Supplementary material for: MYB transcription factor PdMYB118 directly interacts with bHLH transcription factor PdTT8 to regulate wound-induced anthocyanin biosynthesis in poplar
Source: BMC Plant Biol. 2020 Apr 20;20:173. doi: 10.1186/s12870-020-02389-1 (PMC7168848; doi:10.1186/s12870-020-02389-1)
Supplement: Supplementary file 2 — Additional file 2: Figure S2. BiFC assays to test the interaction of PtrJAZ1 with PdMYB118. [file 12870_2020_2389_MOESM2_ESM.docx]

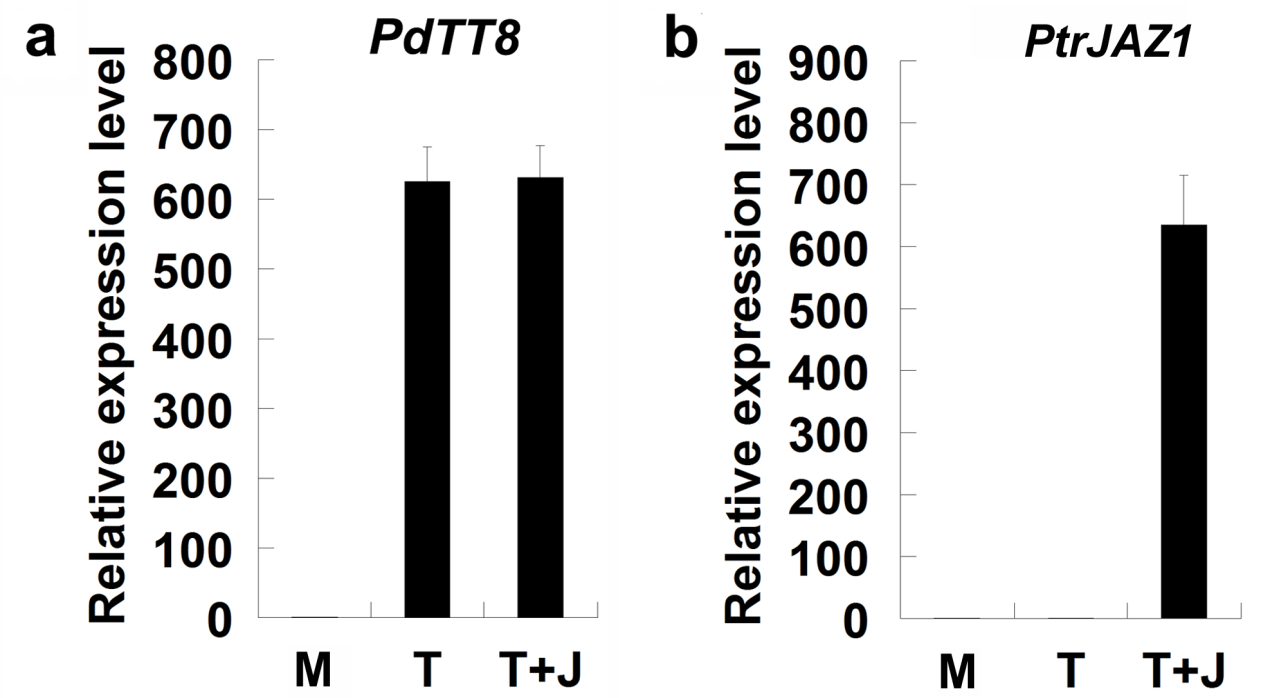


**Figure S2.** Expression of *PtrJAZ1* and *PdTT8* in the protoplasts isolated from the leaves of transgenic plants overexpressing *PdMYB118.* Protoplasts transformed with pGreenII62-SK-*PdTT8* alone or co-transformed with pGreenII62-SK-*PdTT8* and pGreenII62-SK-*PtrJAZ1* were used for the analyses. (a) Expression level of *PdTT8*. (b) Expression level of *PtrJAZ1.* The empty vector was used as a negative control and the gene expression level in the control was set to 1. M, transgenic protoplasts transfected with pGreenII62-SK; T, transgenic protoplasts transfected with pGreenII62-SK-*PdTT8*; T+J, transgenic protoplasts co-transfected with pGreenII62-SK-*PdTT8* and pGreenII62-SK-*PtrJAZ1*.
