# Supplementary material for: MYB transcription factor PdMYB118 directly interacts with bHLH transcription factor PdTT8 to regulate wound-induced anthocyanin biosynthesis in poplar
Source: BMC Plant Biol. 2020 Apr 20;20:173. doi: 10.1186/s12870-020-02389-1 (PMC7168848; doi:10.1186/s12870-020-02389-1)
Supplement: Supplementary file 3 — Additional file 3: Figure S3. Expression of PtrJAZ1 and PdTT8 in the protoplasts isolated from the leaves of transgenic plants overexpressing PdMYB118. [file 12870_2020_2389_MOESM3_ESM.docx]

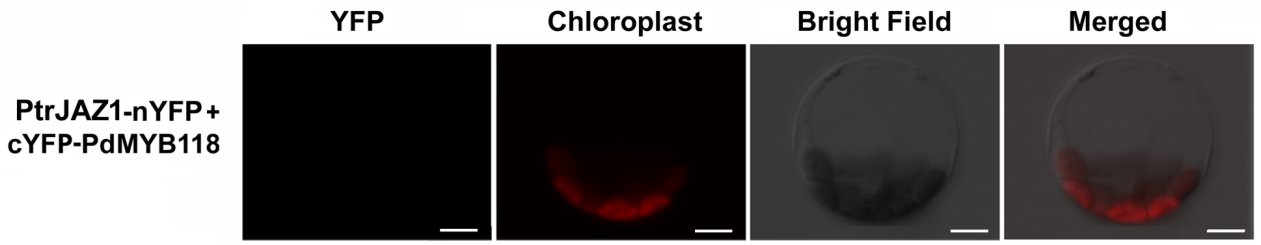


**Figure S3.** BiFC assays to test the interaction of PtrJAZ1 with PdMYB118. PtrJAZ1 was fused with the N-terminal fragment of YFP, and PdMYB118 was linked to the C-terminal fragment of YFP. Construct pairs indicated on the left were co-expressed in poplar leaf protoplasts. Scale bar = 10 μm.
