# Supplementary material for: MYB transcription factor PdMYB118 directly interacts with bHLH transcription factor PdTT8 to regulate wound-induced anthocyanin biosynthesis in poplar
Source: BMC Plant Biol. 2020 Apr 20;20:173. doi: 10.1186/s12870-020-02389-1 (PMC7168848; doi:10.1186/s12870-020-02389-1)
Supplement: Supplementary file 4 — Additional file 4: Figure S4. qRT-PCR analyses of PdMYB118 and PdTT8 in the wounded leaves of WT and transgenic plants overexpressing PdMYB118. [file 12870_2020_2389_MOESM4_ESM.docx]

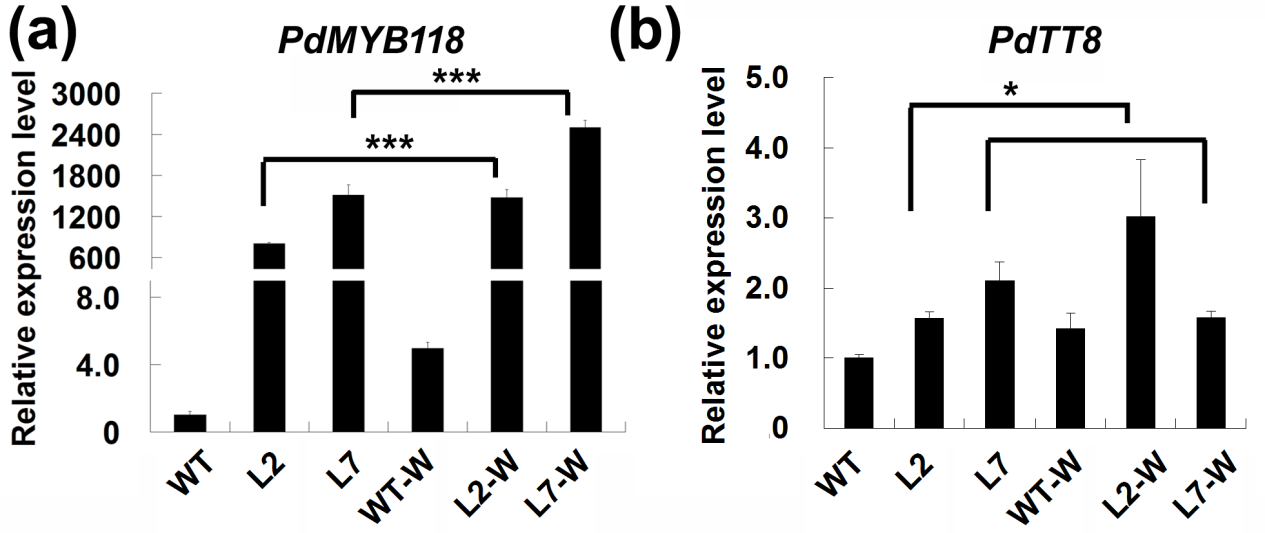


**Figure S4.** qRT-PCR analyses of *PdMYB118* and *PdTT8* in the wounded leaves of WT and transgenic plants overexpressing *PdMYB118.* Gene expression level in the leaves of WT was set to 1. Values are means and standard deviations of three biological replicates (n = 3). * and ***, significant differences at *P* < 0.05 and *P* < 0.001 (Student’s t-test). WT, wide type poplar plant; L2 and L7, transgenic plants overexpressing *PdMYB118*; WT-W, wounded leaves of WT plants; L2-W and L7-W, wounded leaves of transgenic plants
