# Supplementary material for: MYB transcription factor PdMYB118 directly interacts with bHLH transcription factor PdTT8 to regulate wound-induced anthocyanin biosynthesis in poplar
Source: BMC Plant Biol. 2020 Apr 20;20:173. doi: 10.1186/s12870-020-02389-1 (PMC7168848; doi:10.1186/s12870-020-02389-1)
Supplement: Supplementary file 5 — Additional file 5: Figure S5. A proposed model of wound induced anthocyanin biosynthesis in poplar. [file 12870_2020_2389_MOESM5_ESM.docx]

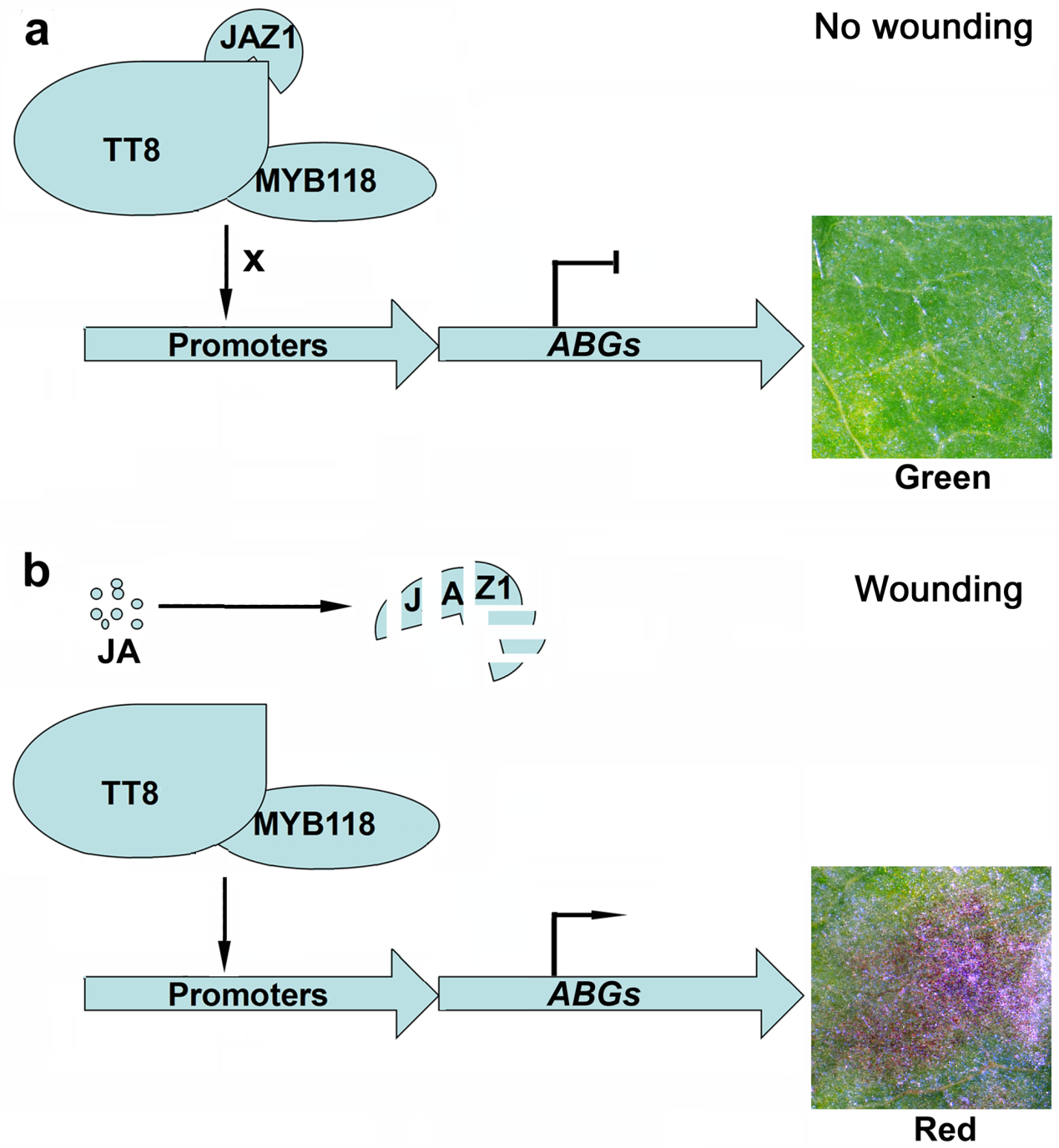


**Figure S5.** A proposed model of wound induced anthocyanin biosynthesis in poplar. (a) Under normal condition, JAZ1 protein binds to the transcription factor TT8 to inhibit the transcription activation activity of MYB118/TT8 complex, the expressions of *ABGs* and the biosynthesis of anthocyanin are repressed. (b) Upon wood induction, JAZ1 protein degradation is induced by the increased JA biosynthesis, the transcription activation activity of MYB118/TT8 complex is released, and the expressions of *ABGs* are activated for anthocyanin biosynthesis.
