# Supplementary material for: MYB transcription factor PdMYB118 directly interacts with bHLH transcription factor PdTT8 to regulate wound-induced anthocyanin biosynthesis in poplar
Source: BMC Plant Biol. 2020 Apr 20;20:173. doi: 10.1186/s12870-020-02389-1 (PMC7168848; doi:10.1186/s12870-020-02389-1)
Supplement: Supplementary file 6 — Additional file 6: Figure S6. Western blotting analyses of JAZ1 protein in the wounded leaves. [file 12870_2020_2389_MOESM6_ESM.docx]

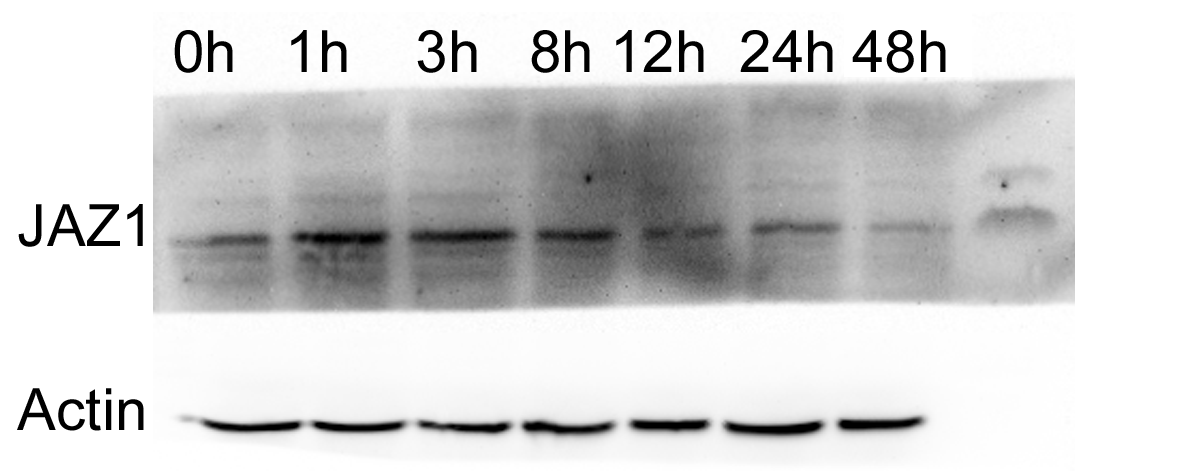


**Figure S6.** Western blotting analyses of JAZ1 protein in the wounded leaves. The original uncropped blot images for Fig. 4d are shown.
