# Supplementary material for: MYB transcription factor PdMYB118 directly interacts with bHLH transcription factor PdTT8 to regulate wound-induced anthocyanin biosynthesis in poplar
Source: BMC Plant Biol. 2020 Apr 20;20:173. doi: 10.1186/s12870-020-02389-1 (PMC7168848; doi:10.1186/s12870-020-02389-1)
Supplement: Supplementary file 8 — Additional file 8: Table S1. Primer sequences used in this study. [file 12870_2020_2389_MOESM8_ESM.docx]

**Supplementary information**

**Table S1.** Primer sequences used in this study.

| **Gene** | **Identifier** | **Forward Primer (5' to 3')** | **Reverse Primer (5' to 3')** |
| --- | --- | --- | --- |
| **RT-PCR primers** | | | |
| *PtrCHS1* | eugene3.00140920 | AAACGTCCTGAGTGATTATGGC | ATTGCTGGTCGAACGATCTTAT |
| *PtrCHI1* | estExt_Genewise1_v1.C_LG_X2396 | CACGAACTTACCTCACCCACC | CGTTAATGGCAATATCATCGTCA |
| *PtrF3H* | gw1.57.31.1 | ATGGAAAGCAGTGACCGAGGA | CTTCAACAGGTTGAACGGTGATC |
| *PtrF3H’* | estExt_fgenesh4_pg.C_LG_XIII0337 | ATGGTGACCCGAAGGCAGAT | GCAATAGCCCATTCCACTGT |
| *PtrF3'5'H2* | eugene3.00011827 | CAAGGAATTGAACGTGGGATG | GCAGGTATGGAAGTTTTGGTATGT |
| *PtrDFR2* | gw1.V.1411.1 | GGATTTTATCAGCGTCATACCACC | AGACAACACTCGCCAAATCCTC |
| *PtrANS1* | grail3.0018022801 | ACTACTACCCCAAGTGCCCTCA | ACAATCTCTGCCAATGGCTTG |
| *PdMYB118* | gw1.II.2535.1 | GCAGGAAAAGCTGTAGAATGAGG | GGAGTTCCTTTTCCAGCTCTTAAC |
| *PdTT8* | gw1.II.1966.1 | TCTTAGTGTGGGGAGATGGGTAT | ATCGTAGGCCTTTCCTGGTAAC |
| *PtrJAZ1* | estExt_Genewise1_v1.C_280165 | ATTTGCTCCCAAGGAAGATGTC | TTGGCTGGAATAGAAGGAAAGG |
| *PtrEF1β* | eugene3.00091463 | GACAAGAAGGCAGCGGAGGAGAG | CAATGAGGGAATCCACTGACACAAG |
| **Primers for gene clone** | | | |
| PdTT8 | gw1.II.1966.1 | ATGCTGCAGGCTGCGGTGC | TCAATCATGGGGTATTATTTGACGTAT |
| *PtrJAZ1* | estExt_Genewise1_v1.C_280165 | ATGTCTGGCTCGACGGAATT | CTAGTGTGTTGTAGATGGAGCAGC |
| **Primers for promoter clone** | | | |
| *PtrCHS1* | eugene3.00140920 | TTTATGTTGCGAGGCCCACTA | TGAAAGACAAATGCAATAAAGAGGT |
| *PtrDFR2* | gw1.V.1411.1 | TAGGAACTTATTAGTGTGGGGGTG | CTGATGATGAGTAGTAACGGGGTC |
| *PtrANS1* | grail3.0018022801 | AAATCTTAAAGGGTATGTGGAGGAA | AAAATCGTTTGATAGGATGCTGAG |
